# Genetic Characterizations and Molecular Evolution of the Measles Virus Genotype B3’s Hemagglutinin (H) Gene in the Elimination Era

**DOI:** 10.3390/v13101970

**Published:** 2021-09-30

**Authors:** Nan Zhou, Mingma Li, Yue Huang, Lu Zhou, Bei Wang

**Affiliations:** 1 Department of Epidemiology and Biostatistics, School of Public Health, Southeast University, Nanjing 210009, China; sduzhounan@163.com (N.Z.); 220203793@seu.edu.cn (M.L.); greataq@163.com (Y.H.); 2 Jiangsu Provincial Center for Disease Control and Prevention, Department of Acute Infectious Diseases, Nanjing 210009, China; zhoulu1212@163.com

**Keywords:** measles virus, genotype B3, hemagglutinin gene, evolution

## Abstract

Measles virus (MeV) genotype B3 is one globally significant circulating genotype. Here, we present a systematic description of long-term evolutionary characterizations of the MeV genotype B3’s hemagglutinin (H) gene in the elimination era. Our results show that the B3 H gene can be divided into two main sub-genotypes, and the highest intra-genotypic diversity was observed in 2004. MeV genotype B3’s H gene diverged in 1976; its overall nucleotide substitution rate is estimated to be 5.697 × 10^−4^ substitutions/site/year, and is slowing down. The amino acid substitution rate of genotype B3’s H gene is also decreasing, and the mean effective population size has been in a downward trend since 2000. Selection pressure analysis only recognized a few sites under positive selection, and the number of positive selection sites is getting smaller. All of these observations may reveal that genotype B3’s H gene is not under strong selection pressure, and is becoming increasingly conservative. MeV H-gene or whole-genome sequencing should be routine, so as to better elucidate the molecular epidemiology of MeV in the future.

## 1. Introduction

Measles is a highly contagious illness manifesting as a characteristic erythematous, maculopapular rash caused by the measles virus (MeV) [1]. In the period 2000–2016, the incidence of measles declined from 145 per 1 million population to 18 per 1 million population. However, in recent years, measles has re-emerged, and its incidence increased to 25, 49, and 120 per 1 million population in the years from 2017 to 2019, respectively [2,3,4]. Measles outbreaks have also been observed in some developed regions with high vaccine coverage [5,6,7]. The WHO’s established goal of eradicating measles is challenging, and measles is still a major public health issue for human beings. Delays in pediatric vaccination as a consequence of the COVID-19 pandemic may exacerbate the transmission of measles and other vaccine-preventable diseases [8].

MeV is a single-stranded negative-sense RNA virus belonging to the *Morbillivirus* genus and the *Paramyxoviridae* family; its genome consists of ~16,000 nucleotides, encoding six structural proteins (nucleoprotein, phosphoprotein, matrix, fusion, hemagglutinin and large protein) and two non-structural proteins [9]. MeV is considered to be a serologically monotypic virus. Twenty-four genotypes are designated based on the nucleotide sequences of the hemagglutinin (H) and nucleoprotein (N) genes, which are the most variable genes in their viral genome [10]. At present, the diversity of circulating MeV genotypes is decreasing. From 2005 to 2014, there were 13 genotypes circulating in the world [1]; from 2018 to 2019, only 4 (B3, D4, D8, and H1) were detected globally [11]; of these, B3 is one important outbreak-associated genotype [12,13,14], and was also found to be significantly more transmissible than other genotypes [15].

All of this reminds us of the need to explore the long-term molecular evolutionary characteristics of MeV genotype B3. Today, sequencing of the 450-nucleotide region of the N gene (N450) is recommended by the WHO. However, sequencing of the H gene—which possesses important immune epitopes, and is the major target of virus-neutralizing antibodies against MeV infection [16]—is not routine. Genetic analysis of the H gene can provide more valuable information about the molecular epidemiology of MeV [17]. Currently, phylogeny, mutants, selection pressure, and evolutionary estimates of MeV B3’s H gene have been partially published. For example, Kimura et al. estimated that B3’s H gene diverged in 1979 [18]. Bianchi et al. described how MeV B3’s H gene has two sub-genotypes. There is a spatial and temporal difference between strains across sub-genotypes and clades, and three mutations (positions 178, 307, and 400) were important in the evolution of sub-genotype 3.2. A clade (400V) has also been identified [19]. Selection pressure analyses performed on all genotypes identified only a few sites under positive selection, and there is partial concordance between the studies (particularly sites 476 and 575) [18,19]. Here, we retrieved all complete sequences of B3’s H gene from GenBank, and further confirmed and updated previous results. In addition, we presented structural features of some key residues, along with the time-evolutionary pattern of MeV genotype B3’s H gene. All of these will expand the understanding of the evolution of MeV genotype B3’s H gene in the elimination era.

## 2. Materials and Methods

### 2.1. Dataset

The complete genome (1854 nt) of MeV genotype B3’s H gene was retrieved from the GenBank database. Strains with an unknown collection year, undetermined nucleotides, or from environmental samples and recombination signals detected by Recombination Detection Program (RDP) version 4.56 [20] were also excluded. 

### 2.2. Genetic and Amino Acid Diversity of MeV Genotype B3’s H Gene

Sequence alignment was performed using BioEdit version 7.1.3.0 [21]. Then, phylogenetic relation of MeV genotype B3’s H gene was constructed via a maximum likelihood (ML) method based on the K2P+G4 model by using MEGA version X, with 1000 bootstrap replications for branch support [22]. To evaluate genetic and amino acid diversity, sequence identities were calculated using BioEdit 7.1.3.0 [21]. Shannon’s entropy values of amino acid positions were also estimated using the web service of the Shannon Entropy-One tool (www.hiv.lanl.gov, accessed on 12 August 2021) to identify sites with greater variability. Selection pressure analysis was carried out on Datamonkey server (http://www.datamonkey.org, accessed on 20 May 2021) via SLAC, FEL, FUBAR, and MEME methods [23]. Significant positive selection (dN > dS) was indicated with a cutoff *p*-value of 0.05, or a posterior probability of 95%. Then, all identified sites with greater variability and positive selection were mapped onto a 3D structure of the H-protein (PDB number: 2ZB6), and PyMOL version 2.3.0 was used for visualization and annotation [24].

### 2.3. Time-Evolutionary Pattern of MeV Genotype B3’s H Gene

To evaluate the fluctuation in the evolutionary rate over time, root-to-tip regression analysis was inferred using TempEst version 1.5, based on the ML phylogenetic tree for each phylogenetic sub-genotype at the nucleotide level [25]. At the amino acid level, the yearly mean differences in the number of amino acids were calculated with reference to the oldest MeV genotype B3 H gene (isolated in 1993) and plotted against strains’ year of collection, and a locally weighted scatterplot smoothing (LOWESS) fit line was also added. To evaluate the temporal fluctuation of intra-genotypic diversity, the mean pairwise *p*-distance of nucleotides and the mean pairwise differences in the number of amino acids within strains collected in the same year were calculated and plotted against time.

### 2.4. Time-Scale Phylogenetic and Phylodynamic Analysis

Time-scale phylogenetic and phylodynamic analysis was conducted using BEAST version 1.8.3, based on the Bayesian Markov chain Monte Carlo (MCMC) method [26]. Briefly, the best fit substitution model was selected by using the IQ-TREE web server (http://iqtree.cibiv.univie.ac.at/, accessed on 3 May 2021) [27]. Three clock models (strict clock, uncorrelated lognormal relaxed clock, and uncorrelated exponential relaxed clock) and three tree priors (Bayesian skyline coalescent, constant size, and exponential growth model) were selected and compared using the Akaike information criterion via MCMC (AICM). As a result, the dataset was analyzed using the K2P+G4 substitution model under a strict clock model and a Bayesian skyline coalescent tree prior (results of model comparison were shown in Table S1). MCMC chains were run for 100 million steps, and Tracer version 1.6 (http://tree.bio.ed.ac.uk/software/tracer/, accessed on 1 March 2021) was used to extract the results of the evolutionary rate, divergence time, and Bayesian skyline plot (BSP) where an effective sample size value greater than 200 was considered acceptable. The maximum clade credibility (MCC) tree was constructed after 10% burn-in by TreeAnnotator version 1.8.2 (http://tree.bio.ed.ac.uk/software/, accessed on 1 March 2021), and visualized using FigTree version 1.4 [28].

## 3. Results

### 3.1. Genetic and Amino Acid Variation of MeV Genotype B3’s H Gene

According to inclusion and exclusion criteria, a total of 205 complete sequences of MeV genotype B3’s H gene were retrieved from the GenBank database, with the isolation time ranging from 1993 to 2019 (Supplementary Table S2). The nucleotide and amino acid identities among these sequences were 96.7–100% and 97.2–100%, respectively. The phylogenetic tree was constructed based on the ML method, revealing two sub-genotypes, named 3.1 and 3.2(Supplementary Figure S1). The sequences of sub-genotype 3.1 were collected from 1993 to 2001, with the nucleotide and amino acid identities in the ranges of 98.2% to 100% and 98.0% to 100%, respectively. The sequences of sub-genotype 3.2 had a longer timespan (1997–2019), and the nucleotide and amino acid identities were both 97.5–100%. The *p*-distance of nucleotides between sub-genotypes 3.1 and 3.2 was 2.40% ± 0.28%, ranging from 1.51% to 3.24%. The frequency of amino acid mutations greater than 50% is summarized in Table 1. Four specific substitutions (240N, 283G, 303D, and 471E) were found to be associated with the lineage of sub-genotype 3.2. In fact, 173 of the 174 sequences with a 471E, 172 of the 173 sequences with a 240N, and all of the sequences with a 283G or 303D belonged to sub-genotype 3.2. Another three substitutions (400V, 178T, and 307I) were found to be related to B3-400V clade branches, which were previously described in [19], and separated sub-genotype 3.2 into non-400V strains (collected from 1997 to 2012) and the 400V clade (collected from 2006 to 2019). The *p*-distance of nucleotides within these two groups was 0.89% ± 0.09% and 0.47% ± 0.08%, respectively. The *p*-distance of nucleotides between these two groups was 1.21% ± 0.16%.

**Figure 1:**
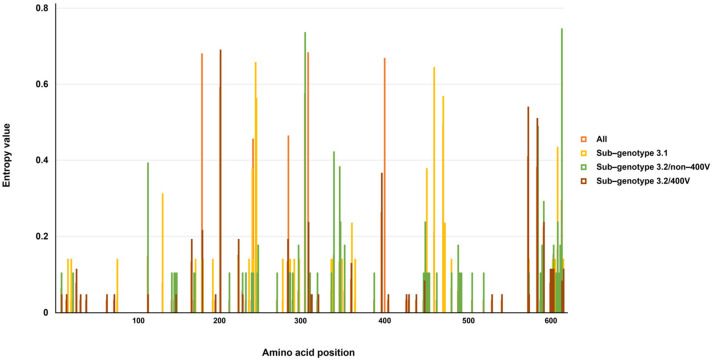
Shannon’s entropy of MeV genotype B3’s H gene for all analyzed strains and different sub-genotypes.

Then, we calculated the entropy value of each amino acid position for all B3 H strains and each sub-genotype, in order to identify the high-variable site, which was defined as having an entropy value greater than 0.6. Our results showed that only few sites were significantly variable (Figure 1). In detail, for all strains, three residues (at positions 178, 307, and 400) were determined. For sub-genotype 3.1, two residues (at positions 243 and 460) were found. For sub-genotype 3.2 non-400V strains and the 400V clade, two residues (at positions 303 and 615) and one position (at position 200) were detected, respectively. Selection analysis was also performed on the MeV genotype B3’s H gene. For all analyzed strains, only one (at position 616) and two sites (at positions 615 and 616) under positive selection were recognized by the FUBAR and MEME methods, respectively. For sub-genotype 3.1, five (at positions 2, 43, 250, 289, and 460), one (at position 460), and two (at positions 460 and 616) sites under positive selection were detected by the FEL, FUBAR, and MEME methods, respectively. For sub-genotype 3.2, only one positive selection site (at position 303) was found by the FEL and FUBAR methods in sub-genotype 3.2/non-400V strains, and no site under episodic diversifying selection was observed in the sub-genotype 3.2/400V clade, by all methods.

**Figure 2:**
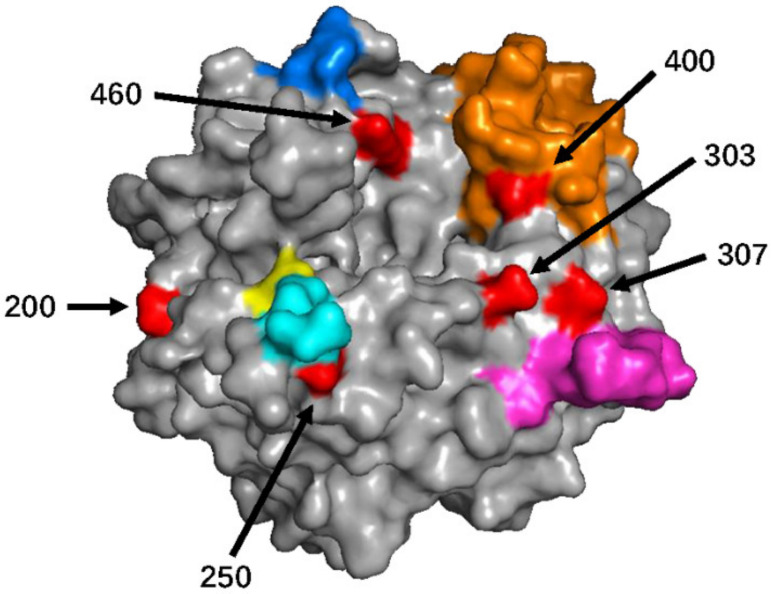
Mapping of the amino acid positions with high variations and positive selection pressure on the structural model of the MeV H protein (PDB number: 2ZB6). The hemagglutinating and neutralizing epitope (HNE), neutralizing epitope (NE), receptor binding epitope (RBE), loop epitope (LE), and sugar-shielded epitope (SSE) are colored in orange, cyan, marine blue, light magenta, and yellow, respectively. Detected amino acid positions with high variations and positive selection pressure are in red (positions 43, 615, and 616 were absent in this model).

Next, all sites with high variation and positive selection pressure were mapped onto the 3D structure of the H protein (2, 43, 178, 615, and 616 were not mapped, because the structural features in the model were absent). Excluding position 289, other sites were surface-exposed (Figure 2). Positions 250 and 400 were mapped on the neutralizing epitope (NE) and the hemagglutinating and neutralizing epitope (HNE), respectively [29].

### 3.2. Time-Evolutionary Pattern of MeV Genotype B3’s H Gene

**Figure 3:**
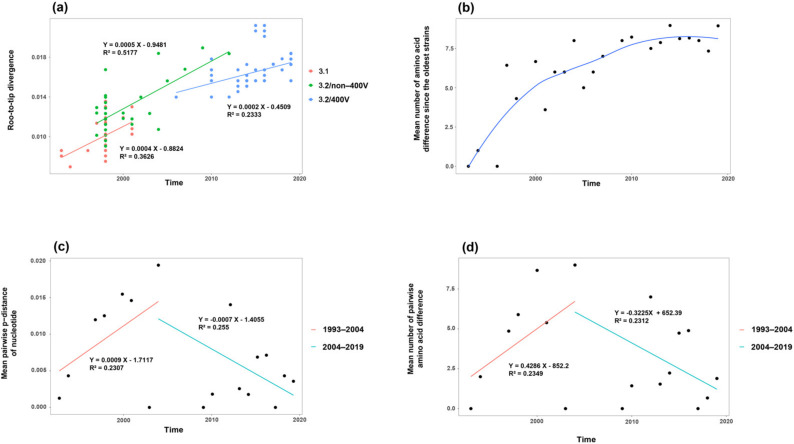
Time-evolutionary patterns of MeV genotype B3’s H gene. (**a**) Root-to-tip divergence plots; (**b**) mean number of amino acid differences since the oldest strains; (**c**) mean *p*-distance of nucleotides within strains collected in the same year; (**d**) mean number of amino acid differences within strains collected in the same year.

In order to evaluate the time-evolutionary pattern of nucleotides on MeV genotype B3’s H gene, we plotted the root-to-tip divergence against the time of sampling of each strain based on the inferred ML tree. Linear regression analysis indicated that all sub-genotypes exhibited a positive correlation between genetic divergence and sampling time, and presented a moderate linear evolution (Figure 3a). The slopes of sub-genotype 3.1 and sub-genotype 3.2/non-400V strains were similar, both of which were greater than the slope of the sub-genotype 3.2/400V clade. Then, the mean number of amino acid differences was calculated yearly with reference to the oldest B3 strain (isolated in 1993), and plotted against strains’ year of collection. The LOWESS fit line was also added (Figure 3b), whose slope decreased with time. In fact, the slope of linear regression analysis was 0.4416 from 1993 to 2009; however, the slope of linear regression analysis was 0.0251 from 2010 to 2019.

We also estimated the mean pairwise *p*-distance of nucleotides and the mean number of pairwise amino acid differences within strains collected in the same year, in order to evaluate the temporal fluctuation of intra-genotypic diversity. The highest intra-genotypic diversity was observed in 2004, and the *p*-distance of nucleotides and the number of amino acid differences were 0.0194 ± 0.0031 and 9 ± 2.8121, respectively. The plots of differences in nucleotides and amino acids against time also indicated that the intra-genotypic diversity increased from 1993 to 2004, with a moderate linear trend, and since then, has been decreasing with a moderate linear trend (Figure 3c,d). There were two points (2003 and 2012) that were outliers and did not fit the linear models. The sequences in 2003 were all from Spain, with high nucleotide identity, and belonged to sub-genotype 3.2/non-400V strains, explaining the low diversity in 2003. The sequences in 2012 belonged to sub-genotype 3.2 non-400V strains and the 400V clade. The co-circulation of two sub-genotypes resulted in the high diversity in 2012.

### 3.3. Time-Scale Phylogenetic and Phylodynamic Analysis

**Figure 4:**
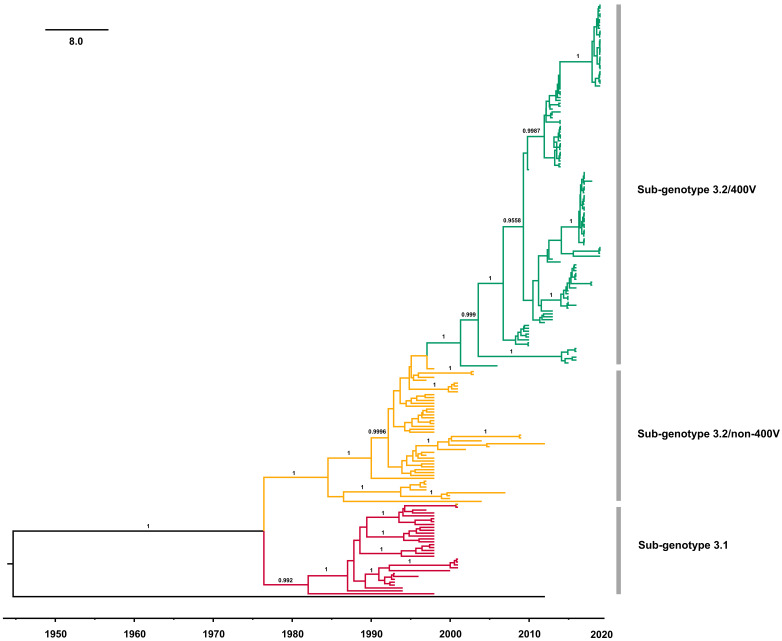
Phylogenetic tree of MeV genotype B3’s H gene, constructed by the Bayesian Markov chain Monte Carlo method. The scale bar represents the unit of time (year), and the divergence times of sub-genotypes were added. The sequence from genotype D4 (MVi/California, USA/16.12) was used as an outgroup.

**Figure 5:**
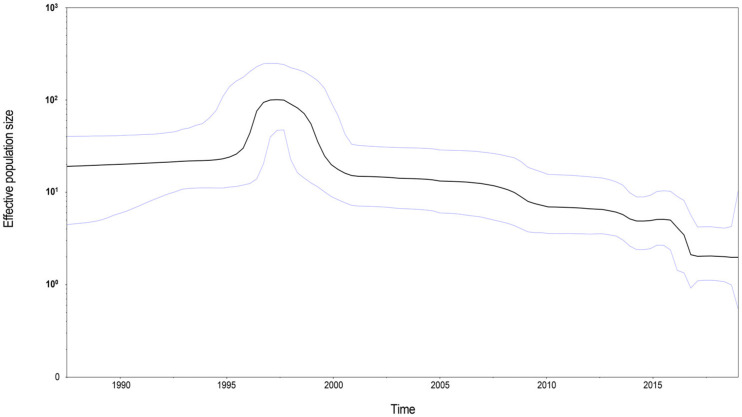
Bayesian skyline plot of MeV genotype B3’s H gene for all selected strains. The *Y*-axis and *X*-axis indicate the effective population size and the time (year), respectively. Mean effective population is displayed as a black line. The 95% highest posterior densities are shown as light blue lines.

A time-scale phylogenetic tree was constructed using the Bayesian MCMC method with the nucleotide sequences of the MeV genotype B3’s H gene (Figure 4). The results showed that MeV genotype B3’s H gene diverged in 1976 (95% highest posterior densities (HPDs): 1970–1982). The years of divergence of sub-genotype 3.2 non-400V strains and the 400V clade were 1984 (95% HPDs: 1980–1989) and 1997 (95% HPDs: 1995–1998), respectively. Then, the overall nucleotide evolutionary rate of MeV genotype B3’s H gene was estimated to be 5.697 × 10^−4^ substitutions/site/year (95% HPDs: 4.105 × 10^−4^–7.325 × 10^−4^ substitutions/site/year). For each sub-genotype, the molecular evolution rates of sub-genotype 3.1 and the sub-genotype 3.2/non-400V strains were similar, and estimated to be 1.071 × 10^−3^ substitutions/site/year (95% HPDs: 5.254 × 10^−4^–1.602 × 10^−3^ substitutions/site/year) and 8.090 × 10^−4^ substitutions/site/year (95% HPDs: 4.160 × 10^−4^–1.238 × 10^−3^ substitutions/site/year), respectively. The sub-genotype 3.2/400V clade had a lower evolutionary rate of 4.870 × 10^−4^ substitutions/site/year (95% HPDs: 2.940 × 10^−4^–6.839 × 10^−4^ substitutions/site/year). Then, a BSP was constructed to evaluate the demographic history of MeV genotype B3’s H gene (Figure 5). The mean effective population size of MeV genotype B3’s H gene presented a peak around 1997, and began to descend slowly after 2000. After 2015, a sharp fall was observed, and the mean effective population size of MeV genotype B3’s H gene has remained stable since then.

## 4. Discussion

B3 is a frequently detected circulating MeV genotype globally. In this study, we reported a comprehensive molecular characterization of MeV genotype B3’s H gene based on the full strains. The 2.5% and 2.0% nucleotide divergences in the MeV N and H genes, respectively, were used to define a new MeV genotype by the WHO criteria [30]. In this study, the results of phylogenetic analysis revealed two main sub-genotypes (3.1 and 3.2) of MeV B3’s H gene, as reported previously [19,31,32], and the minimum genetic distance between the proposed B3 sub-genotypes (1.51%) did not match the 2.0% diversity threshold for assigning a new genotype. Based on the N450 gene, MeV B3 can be divided into three lineages [33,34]. In our analysis, sub-genotype 3.2 can be further divided into non-400V strains and a 400V clade. The collection time also revealed that these three clades circulated almost alternately, and that the sub-genotype 3.2/400V clade was the only variant circulating globally in the past decade. Similar results were also observed by Bianchi et al. [19]. In a comparison of neutralizing antibody titers against outbreak-associated measles genotypes (D4, H1, and B3) in Iran, the lowest geometric mean titer of antibodies against B3 was observed [35]. The strain of B3’s H gene (accession number: MH922967) in that comparative study was included in our analysis, and belonged to the sub-genotype 3.2/400V clade. This may indicate that the effectiveness of vaccines against the sub-genotype 3.2/400V clade should be evaluated in regions where this sub-genotype has been frequently recognized.

Next, we identified the amino acid signatures of the sub-genotypes. There were fewer amino acid residues responsible for the distinction of the sub-genotypes, and we speculate that the evolution of MeV’s H gene may only be promoted by some key sites. These results are consistent with those of previous studies [19,32]. Furthermore, the sub-genotype 3.2/400V clade was the only circulating lineage in the past decade. Thus, substitutions of 400V, 178T, and 307I in this clade may suggest the spread of the virus. Selection analysis revealed that B3’s H protein was well conserved, and displayed fewer sites under positive selection pressure. Most of the sites under positive selection were inconsistent with previous reports, except for sites 303 and 616; this may be because of the differences in the datasets and methods of analysis. In addition, sites 303 and 616 may be responsible for the regulation of interaction between MeV’s H protein and CD46 [36]. More interestingly, we found that the number of sites under positive selection pressure tended to become smaller from sub-genotype 3.1 to sub-genotype 3.2/400V. This may suggest that B3’s H protein is becoming increasingly conservative. Then, we compared the sites of amino acid signatures among sub-genotypes and sites under high variation or positive selection pressure with the known antigen epitopes. Most of these residues were not important for the binding receptors SLAM, CD46, and nectin-4 [37,38], and fewer residues were located at the epitopes. Others were mapped on the surface near antigenic sites; of these, position 200 is an N-linked glycosylation site and part of the SSE, covering a wide area of its surface on MeV’s H protein and limiting the areas available for antibody binding [39,40]. Position 200 (N/D) was found to be a high-variable residue in the sub-genotype 3.2/400V clade. Whether this substitution has an enhanced potential for vaccine breakthrough of this clade remains to be explored.

The LOWESS fit line to discuss the evolutionary fluctuation at the amino acid level indicated that the mean number of amino acid differences since the oldest strains was increasing, but the accumulation rate was slowing down, revealing that the substitution rate of amino acids was decreasing. This result was similar to root-to-tip regression analysis by sub-genotypes, which was used to discuss the time-evolutionary pattern at the nucleotide level. The evolutionary characterizations of viruses may be time-dependent due to the effect of natural selection [41]. Positive selection may increase the overall substitution rate for an adaptive molecular evolution, and negative selection may indicate a decline in the rate of evolution. Therefore, the decreasing evolutionary rate of MeV genotype B3’s H gene may be the result of the selection pressure becoming smaller, as previously mentioned. 

Expanded immunization activities have led to a significant reduction in the genotype diversity of MeV [3]. The intra-genotypic diversity of specific MeV genotypes has also been influenced. For example, the decline in the genetic diversity of MeV D6’s N450 gene in Europe and H1’s N450 gene in China may be attributed to enhanced vaccination [42,43]. Our results showed that there was a greater temporal variation in intra-genotypic diversity, but no obvious upward or downward trend of intra-genotypic diversity was observed. The limited number of analyzed strains in each year may prevent us from recognizing the real variation pattern of intra-genotypic diversity of MeV genotype B3’s H gene. In addition, although the highest diversity was observed in 2004, and the lower yearly diversity of B3’s H gene was observed at both the nucleotide and amino acid levels in recent years, this may be an artifact of the fact that sequencing is becoming cheaper, and more sequences were from the same infection clusters.

We estimated that B3 diverged in 1976, which was approximately consistent with previous estimates [18]. The evolutionary rates of MeV’s H gene in different genotypes are variable, ranging from 7.28 × 10^−6^ substitutions/site/year to 6 × 10^−3^ substitutions/site/year [44,45,46,47], and the overall evolutionary rate of the H gene in all MeV genotypes was estimated to be 9.02 × 10^−4^ substitutions/site/year [18]. We estimated that the substitution rate of B3’s H gene was only 5.697 × 10^−4^ substitutions/site/year. The estimate of the evolutionary rate of each sub-genotype also found that sub-genotypes 3.1 and 3.2a exhibited a similar rate, while sub-genotype 3.2b had the slowest evolutionary rate, as shown by root-to-tip divergence plots. Additionally, compared with the evolutionary rate of the N gene as described previously [48], the H gene evolves more slowly. The phylogenetic relationships of the N gene can be clustered according to the H gene [49], and the H and N genes can provide different information on intra-genotypic diversity [17]. Thus, complete genome sequencing should be promoted in the surveillance of MeV in order to increase the molecular resolution in the future.

Effective population size reflects sequence variability, and can elucidate a demographic history during a time interval [50]. In this study, BSP analysis showed that MeV genotype B3’s H gene experienced expansion dynamics from 1995 to 2000, and then declined over the past two decades, which is contrary to the higher detection rate of B3 globally in recent years. Currently, vaccination activities have reduced the number of circulating MeV genotypes, and the higher detection rate of B3 may be a result of the increase in its relative proportion when compared with other genotypes.

In conclusion, sequencing of MeV’s H gene is not a standard practice within molecular surveillance of measles, and analyzed strains may be biased, since virological surveillance is temporally and geographically uneven. The number of analyzed strains is still relatively small, and there is a lack of comparison with other MeV genotypes in this study. The absence of information on the origins of sequences (e.g., vaccinated vs. unvaccinated individuals) made further analysis difficult. Nevertheless, we still found that the number of sites under positive selection pressure is becoming smaller. The nucleotide substitution rate and amino acid accumulation rate of B3’s H gene are also decreasing. These results may indicate that B3’s H gene is not under strong selection pressure, and is becoming increasingly conservative. Finally, although the control measures to prevent COVID-19 (such as travel restrictions, physical distancing, and wearing masks) have reduced the spread of MeV, measles vaccinations and surveillance have also been delayed [51]. In the future, we should pay more attention to the effect of COVID-19 on measles, and MeV H-gene sequencing or whole-genome sequencing should be strengthened in measles surveillance for a better evaluation of molecular characterizations of MeV.

## Figures and Tables

**Table 1:** Frequency of amino acid mutations.

Mutation	Frequency in All Analyzed B3 Strains	Frequency in Specific Cluster
471 E	84.88% (174/205)	Sub-genotype 3.2: 99.43% (173/174)
240 N	84.39% (173/205)	Sub-genotype 3.2: 99.42% (172/173)
283 G	83.90% (172/205)	Sub-genotype 3.2: 100% (172/172)
303 D	76.10% (156/205)	Sub-genotype 3.2: 100% (156/156)
400 V	61.46% (126/205)	Sub-genotype 3.2/400V: 100% (126/126)
178 T	58.54% (120/205)	Sub-genotype 3.2/400V: 94.44% (119/120)
307 I	57.56% (118/205)	Sub-genotype 3.2/400V: 100% (118/118)

## Supplementary Materials

The following are available online at https://www.mdpi.com/article/10.3390/v13101970/s1: Table S1: Results of model comparison; Table S2: Complete sequences of MeV genotype B3’s H gene, as analyzed in this study; Figure S1: Phylogenetic tree of MeV genotype B3’s H gene, based on maximum likelihood method.
