# Supplementary material for: Genetic Characterizations and Molecular Evolution of the Measles Virus Genotype B3’s Hemagglutinin (H) Gene in the Elimination Era
Source: Viruses. 2021 Sep 30;13(10):1970. doi: 10.3390/v13101970 (PMC8540759; doi:10.3390/v13101970)
Supplement: Supplementary file 1 [file viruses-13-01970-s001.zip › viruses-1361199.pdf]

Table S1 Results of model comparison

Clock model	Tree prior	AICM	S.E.
Strict clock	Bayesian skyline coalescent	12762.557	+/- 0.274
Uncorrelated lognormal relaxed clock	Bayesian skyline coalescent	17782.659	+/- 0.318
Uncorrelated exponential relaxed clock	Bayesian skyline coalescent	17683.957	+/- 0.156
Strict clock	Constant size	12879.323	+/- 0.299
Uncorrelated lognormal relaxed clock	Constant size	17858.05	+/- 0.7
Uncorrelated exponential relaxed clock	Constant size	17829.817	+/- 0.407
Strict clock	Exponential growth model	12790.245	+/- 0.177
Uncorrelated lognormal relaxed clock	Exponential growth model	17806.364	+/- 0.345
Uncorrelated exponential relaxed clock	Exponential growth model	17703.76	+/- 0.159

Lower AICM values indicate better model fit.

Table S2 Complete sequences of MeV genotype B3 H gene analyzed in this study

Accession number	Country	Collection year
AF484955	GMB	1993
AF484954	GMB	1993
EU332936	GMB	1993
EU332939	GMB	1993
L46752	USA	1994
AF484953	FRA	1994
AY059391	GMB	1996
AJ239173	NIE	1997
AF453430	SUD	1997
AF453431	SUD	1997
AJ239134	NIE	1997
AJ239133	NIE	1997
HM439386	SUD	1997
MG912590	SUD	1997
AJ239157	NIE	1998
AJ239146	NIE	1998
AJ239149	NIE	1998
AJ239154	NIE	1998
AJ239141	NIE	1998
AJ239151	NIE	1998
AJ239135	NIE	1998
AJ239175	GHA	1998
AJ239176	GHA	1998
AJ239177	GHA	1998
AJ239145	NIE	1998
AJ239148	NIE	1998
AJ239163	NIE	1998
AJ239147	NIE	1998
AJ239170	NIE	1998
AJ239153	NIE	1998
AJ239171	NIE	1998
AJ239156	NIE	1998
AJ239140	NIE	1998
AJ239158	NIE	1998
AJ239139	NIE	1998
AJ239169	NIE	1998
AJ239168	NIE	1998
AJ239142	NIE	1998
AJ239162	NIE	1998
AJ239159	NIE	1998

AJ239161	NIE	1998
AJ239166	NIE	1998
AJ239164	NIE	1998
AJ239152	NIE	1998
AJ239172	NIE	1998
AJ239165	NIE	1998
AJ239143	NIE	1998
AJ239167	NIE	1998
AJ239150	NIE	1998
AJ239137	NIE	1998
AJ239136	NIE	1998
AJ239155	NIE	1998
AJ239160	NIE	1998
AJ239144	NIE	1998
AJ239138	NIE	1998
AJ239174	GHA	1998
AF480469	DEU	2000
AF453432	SUD	2000
AF453433	SUD	2000
AY159459	BFA	2001
AY159460	BFA	2001
AY159461	BFA	2001
AY159462	BFA	2001
AY159463	BFA	2001
AY159464	BFA	2001
AF484952	CAE	2001
AF484951	CAE	2001
AF484950	CAE	2001
AF484949	CAE	2001
FN594773	TUN	2002
FJ865562	SPA	2003
FJ865561	SPA	2003
DQ267505	FRA	2004
DQ267504	FRA	2004
JN635408	USA	2005
KP191044	SPA	2006
FN594772	LBY	2007
FN594725	TUN	2009
FN594724	LBY	2009
JQ417683	DEU	2010
KC305669	ZAF	2010
KC305668	ZAF	2010
KC305667	ZAF	2010

KC305666	ZAF	2010
KC305665	ZAF	2010
KC305664	ZAF	2010
KC305670	ZAF	2010
KC305663	ZAF	2010
MH922967	IRN	2012
KT732214	GBR	2012
KF672752	CAN	2013
KF704001	CAN	2013
MH920633	IRN	2013
MH918856	IRN	2013
KT732217	GBR	2013
KT732216	GBR	2013
KT732215	GBR	2013
MN630023	KOR	2013
KT851538	ZAF	2014
KP881504	TWN	2014
KP881503	TWN	2014
KP881502	TWN	2014
KP881501	TWN	2014
KP881500	TWN	2014
KP881499	TWN	2014
LC155950	JPN	2014
LC155949	JPN	2014
LC154867	JPN	2014
LC154866	JPN	2014
LC154865	JPN	2014
LC154864	JPN	2014
LC154863	JPN	2014
LC154862	JPN	2014
LC154861	JPN	2014
KY969481	USA	2014
KY969478	USA	2014
KY969477	USA	2014
KT732224	GBR	2014
KT732223	GBR	2014
KT732222	GBR	2014
KT732221	GBR	2014
KT732220	GBR	2014
KT732219	GBR	2014
KT732218	GBR	2014
MK628318	ITA	2015
MK628314	ITA	2015

MK628313	ITA	2015
MK628312	ITA	2015
MK628311	ITA	2015
MK628309	ITA	2015
MK628308	ITA	2015
MK628305	ITA	2015
KX838946	FRA	2016
MK628307	ITA	2016
MK628317	ITA	2016
MK628316	ITA	2016
MK628315	ITA	2016
MK628310	ITA	2016
MK628306	ITA	2016
MK628304	ITA	2016
MK628303	ITA	2016
MK628302	ITA	2016
MK628301	ITA	2016
MK628300	ITA	2016
MK513622	ITA	2017
MK513621	ITA	2017
MK513620	ITA	2017
MK513619	ITA	2017
MK513618	ITA	2017
MK513617	ITA	2017
MK513616	ITA	2017
MK513615	ITA	2017
MK513614	ITA	2017
MK513613	ITA	2017
MK513612	ITA	2017
MK513611	ITA	2017
MK513610	ITA	2017
MK513609	ITA	2017
MK513608	ITA	2017
MK513607	ITA	2017
MK513606	ITA	2017
MK513605	ITA	2017
MK513604	ITA	2017
MK513603	ITA	2017
MK513602	ITA	2017
MK513601	ITA	2017
MK513600	ITA	2017
MK513599	ITA	2017
MK513598	ITA	2017

MN893225	FRA	2018
MN052912	FRA	2018
MN052911	FRA	2018
MT789820	USA	2019
MT789819	USA	2019
MT789818	USA	2019
MT789817	USA	2019
MT789816	USA	2019
MT789815	USA	2019
MT789814	USA	2019
MT789813	USA	2019
MT789812	USA	2019
MT789811	USA	2019
MT789810	USA	2019
MT789809	USA	2019
MT789808	USA	2019
MT789807	USA	2019
MT789806	USA	2019
MT789805	USA	2019
MT789804	USA	2019
MT789803	USA	2019
MT789802	USA	2019
MT789801	USA	2019
MT789800	USA	2019
MT789799	USA	2019
MT789798	USA	2019
MT789797	USA	2019
MT789796	USA	2019
MT789795	USA	2019
MT789794	USA	2019
MT789793	USA	2019
MT789792	USA	2019
MT789791	USA	2019
MT789790	USA	2019
MT789789	USA	2019
MT789788	USA	2019

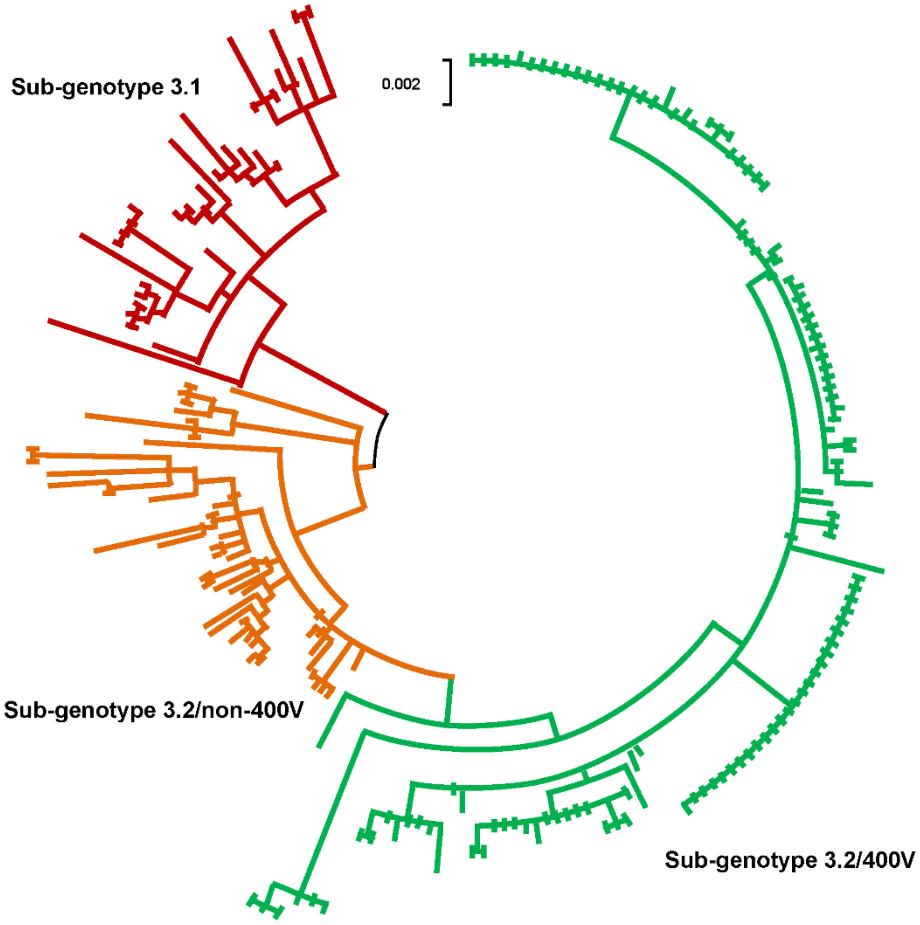

Figure S1. Phylogenetic tree of MeV genotype B3 H gene based on maximum likelihood method**.**
